# Women on the frontline of Covid-19: understanding local women village health volunteers in the northern province of Thailand

**DOI:** 10.1186/s12913-023-09305-x

**Published:** 2023-03-28

**Authors:** Rangsan Sukhampha, Prin Khwanriang, Krisana Vaisamruat

**Affiliations:** 1 Faculty of Sociology, Bielefeld University, X Building, Universitätsstraße 25, 33615 Bielefeld, Germany; 2 Department of Public Administration, Faculty of Humanities and Social Sciences, Valaya Alongkorn Rajabhat University, Pathum Thani, 12120 Thailand; 3 Faculty of Humanities and Social Sciences, Thaksin University, 90000 Songkhla Province, Thailand; 4 Faculty of Political Science, Ramkhamhaeng University, Bangkok, 10240 Thailand

**Keywords:** Local women empowerment, Village Health Volunteer, Community Healthcare, COVID-19, Chiang Mai, Thailand

## Abstract

**Background:**

This paper investigates the role of local women village health volunteers, women on the frontline, during COVID-19 in the northern province of Thailand.

**Methods:**

This research employs a qualitative method with grounded-based analysis of primary data from in-depth interviews of 40 local women village health volunteers that were selected by a purposeful sampling of 10 key informants per district, live in 4 sub-districts in Chiang Mai, the northern province of Thailand: Suthep Subdistrict, Mae Hia Subdistrict, Fa Ham Subdistrict, and Tha Sala Subdistrict.

**Results:**

The role of local women village health volunteers during COVID-19 is diverse, such as community health caregivers, the Surveillance and Rapid Response Team (SRRT), health facilitators and mediators, and the manager of community health funds and resources mobilization. Volunteering for local women in community health services at the local level, participating based on personal desire and foreseeable opportunities, could create meaningful participation for the local women in terms of empowering them and as a driver of local community (health) development.

**Conclusions:**

Findings reveal that understanding local women’s perspectives on their roles could be made through the lens of the intersection of femininity, social role, motivation, and their contribution to their community.

## Background

The global population of women is more than half of the world’s population. However, women’s political and social space in many countries is often overlooked, obscured, and underrepresented, whether it is the dimension of having the right to vote, taking a position of a political leader, or being a high-ranking government official. In international societies, gender equality agreements appear fundamental to international development goals and are seen as a driver of human development. It is crucial to determine the proportion of women in political decision-making. It is not just about equality but also the inclusion of women’s roles in the policy agenda decision-making and change processes and achieving development goals.

The International Declaration of Human Rights supports the right of everyone to participate in public decision-making and activities. The International Convention on the Elimination of All Forms of Discrimination against Women (CEDAW) was promulgated in 1979 by the UN General Assembly, which clearly stated women’s rights in political participation. This is identified in Article 7 states that “States should take appropriate measures to eliminate discrimination against women in the country’s politics and public activities. Women, in particular, must be treated on an equal footing with men” [1]. For example, the right to vote, participation in public policymaking at all levels, participation in public and political NGOs [2], and increasing women’s political participation can also operate through specific training programs [3]. The current COVID-19 pandemic has revealed the extraordinary role women play in public participation, especially the role of volunteering in their communities in various roles such as a role in the food supply, community-kitchen management for those who are hard-pressed and affected by the pandemic crisis, including the role of volunteering as village health volunteers (VHVs). The World Health Organization hailed it as a success factor early in the first pandemic wave in March 2020.

The trend of strengthening women political participation and development occurring around the world and the pandemic of COVID-19 has seen more roles and participation. The researchers focused on the areas affected by the pandemic in Chiang Mai, a major city in the north, and the top 5 tourist destinations of the country, especially tourist attractions in ​​Mueang Chiang Mai District, Chiang Mai Province. This is seen as an area at risk of spreading COVID-19, which is considered a significant target area for the study because there is a risk of super spreader if lax, neglected, strict surveillance and attention. The study targeted local women in Chiang Mai. This means women who live primarily live in the local community, born or from internal migration, and living in the community for a period of time and have a connection and a sense of belonging in the community where they live. VHVs play several roles in the local community, such as a woman, a mother, and a wife, and even all of these simultaneously at the same time as being a volunteer in their villages. This research looks at local women as a discourse to create political meaning and space to play their role in healthcare education for their local communities, especially in the case of the COVID-19 pandemic in Chiang Mai. Its feminine nature, as it is subtle and gentle, would fit well with the nature of work that requires care and trustfulness to patients and people in the community, which create a sense of cooperation with the village health volunteers (VHVs) in community healthcare.

As the report of the public health information system indicates that the VHV population of 2019 within 16 sub-districts in Mueang Chiang Mai district, Chiang Mai province accounted for 2,691 VHVs. This comprises 450 males and 2,241 females in total, representing a female proportion of 83.28% [4]. From the above statistics, it is significant and interesting to investigate further. In particular, the meaningful participation of local women in community healthcare and strengthening community cooperation in preventing and controlling the pandemic.

The paper would contribute to understanding women on the frontline of COVID-19 and provide an essential guideline for increasingly empowering local women to play their roles and mobility. This is to understand and re-interpret the empowerment and release of the shackles of local women’s roles and movements in the traditional context that benefit the community healthcare research and can expand to other issues related to strengthening women’s roles and not being overshadowed and limited as they are ongoing. The paper aims to understand the roles of local women VHVs, their participation in community healthcare during COVID-19, and the meaningful contribution of the participation.

### Frontline health worker, women empowerment, and intersectional perspectives

In general, frontline health workers include all types of health workers who provide care directly to their communities, e.g., nurses, midwives, community health workers, doctors, and pharmacists [5]. Recent works on frontline health workers and women’s empowerment focus on various topics, such as investing in the health workforce for women’s economic empowerment [6], addressing the gender challenges of the health workforce as key to achieving universal health coverage by 2030 [7], gender equity in the health workforce [8], frontline healthcare workers experiences and challenges during the COVID-19 pandemic [9]. In the pandemic context, Women Political Leaders (WPL) asserted that women leaders in healthcare have already demonstrated some of the best responses to COVID-19 worldwide [10]. Most works of literature understand the role of women in healthcare through the lens of intersectionality that encouraged us to move beyond single or typically favoured categories of analysis to consider simultaneous interactions between different aspects of social identity in terms of systems and processes of oppression and domination [11]. Our approach to understanding the roles of local women in frontline healthcare during the pandemic focuses on the intersection of femininity, social role, motivation, and their contribution to their community. In this paper, our focus is specifically on community health workers (CHWs), namely local women village health volunteers (VHVs), who participated in community health care service in case of the Covid-19 pandemic that may create meaningful participation for local women and their community in several ways.

## Methods

To study the role of local women VHVs in enhancing community participation in the prevention, surveillance, and control of the pandemic of COVID-19 in Chiang Mai. The qualitative research method with grounded-based analysis of primary data retrieved from an in-depth interview is employed in the following details.

### Participants

40 local women VHVs were selected by purposeful sampling, classified by locations situated in 4 sub-districts, urban communities, and important tourist attractions in Chiang Mai, consisting of 10 local women VHVs in each sub-districts: Suthep Sub-District Mueang, Mae Hia Sub-district, Fa Ham Subdistrict, and Tha Sala Subdistrict. Regarding our intersectional analytical approach to femininity, social role, motivation, and their contribution to their community, we conducted semi-structured interviews using 5 parts/dimensions of questions that adhere to the logic of intersectional perspectives: (1) key informant general information; (2) background and motivation; (3) participation in community healthcare; (4) problems and obstacles and impacts; and (5) experience acquired and femineity working as a VHV. Each interview lasted 40–60 min and was transcribed. For this research, informed consent was obtained from all subjects and/or their legal guardian(s).

### Data collection and analysis

The paper conducts in-depth interviews to collect information about the role of local women village health volunteers in enhancing community participation in the prevention, surveillance, and control of the pandemic of COVID-19 in Chiang Mai. According to the number specified in the selection process, 40 key informants were interviewed. This project has conducted from 20 to 2020 to 30 March 2021. Data analysis and synthesis were derived from in-depth interviews; the authors also used content analysis, i.e., physical data organization, storage, organizing information content, grouping of data, and conclusion and interpretation. We attempt to understand the local women’s roles in community healthcare service as a volunteer and meaningful participation with grounded-based analysis of the primary data from the fieldwork.

**Table 1:** Characteristics of key informants

Coding	Location (subdistrict)	Years in services	Education Level	Occupation
VHV-101	Suthep	19	Master’s Degree	Business Owner
VHV-102	Suthep	13	High School	Seamstress
VHV-103	Suthep	9	Bachelor’s Degree	Housewife
VHV-104	Suthep	19	Primary School	Governmental Officer
VHV-105	Suthep	22	Primary School	Informal Worker
VHV-106	Suthep	15	Senior High School	Informal Worker
VHV-107	Suthep	19	Junior High School	Informal Worker
VHV-108	Suthep	15	Senior High School	Informal Worker
VHV-109	Suthep	12	Senior High School	Informal Worker
VHV-110	Suthep	23	Junior High School	Informal Worker
VHV-201	Mae Hia	42	Bachelor’s Degree	Member Of the Municipal Council
VHV-202	Mae Hia	28	Senior High School	Administrative Staff (Village Headman)
VHV-203	Mae Hia	6	Senior High School	Thai Traditional Masseuse
VHV-204	Mae Hia	26	Primary School	Administrative Staff (Assistant to the Village Headman)
VHV-205	Mae Hia	18	Junior High School	Informal Worker
VHV-206	Mae Hia	15	Senior High School	Informal Worker
VHV-207	Mae Hia	21	Senior High School	Informal Worker
VHV-208	Mae Hia	13	Junior High School	Informal Worker
VHV-209	Mae Hia	16	Junior High School	Informal Worker
VHV-210	Mae Hia	15	Senior High School	Informal Worker
VHV-301	Fa Ham	38	Bachelor’s Degree	Business Owner
VHV-302	Fa Ham	10	Bachelor’s Degree	Business Owner
VHV-303	Fa Ham	10	Bachelor’s Degree	Vegetable Farmer
VHV-304	Fa Ham	22	Bachelor’s Degree	Pensioner (Former Governmental Officer)
VHV-305	Fa Ham	10	Elementary School (Grade 7)	Seamstress
VHV-306	Fa Ham	16	Senior High School	Informal Worker
VHV-307	Fa Ham	12	Diploma	Informal Worker
VHV-308	Fa Ham	15	Senior High School	Informal Worker
VHV-309	Fa Ham	17	Senior High School	Informal Worker
VHV-310	Fa Ham	21	Primary School	Informal Worker
VHV-401	Tha Sala	17	Bachelor’s Degree	Village Headman
VHV-402	Tha Sala	12	Vocational Certificate	Thai Traditional Masseuse
VHV-403	Tha Sala	19	High School	Business Owner
VHV-404	Tha Sala	2	Bachelor’s Degree	Business Owner
VHV-405	Tha Sala	18	Primary School	Informal Worker
VHV-406	Tha Sala	17	Junior High School	Informal Worker
VHV-407	Tha Sala	14	Senior High School	Informal Worker
VHV-408	Tha Sala	21	Senior High School	Informal Worker
VHV-409	Tha Sala	16	Senior High School	Informal Worker
VHV-410	Tha Sala	18	Junior High School	Informal Worker

## Results

### Characteristics of local women village health volunteers

Several essential data from the fieldwork indicate the meaningful relationship in the role of women’s VHVs participation in local community development and community health care. In this research, key informants were born and grown up, settled, started a family, married locally, or inhabited residence in the area for many years. And they gradually employ a sense of belonging to be a part of the community. They also attained different socioeconomic statuses, which provides different explanations from the existing general perception that local women VHVs are generally viewed as a ‘local woman’ who is slightly “low profile and less educated women.“ A negative meaning and feelings reflect the state of local women being oppressed by norms, values ​​, and culture of Thai society, and those people probably had a low education/knowledge. They were most likely “housewives,“ a term that describes their dependence on men and the burden of household chores, caring for children and husbands simultaneously.

However, data from the fieldwork suggests that the above-mentioned is misunderstood. It is a view of traditional Thai masculinity society where men dominate and suppress and freeze women’s roles. The key informants’ characteristics (see Table 1) reveal the diversity of local women VHVs’ backgrounds, ranging from elementary school to master’s degree (VHV-101), private company employees (see Table 1), government officials (VHV-104), administrative staff (VHV-202, 204), Thai traditional masseuse (VHV-203, 402), business owners (VHV-101, VHV-301-303, VHV-403-404) and local politicians (VHV-201). Years in service as VHVs are between 2 years to 42 years. The informants discussed the reasons and motivations for taking on the role of village health volunteers (VHVs) born out of a love of volunteer work or from surrounding networks and solicitations from community leaders or acquaintances and intended to develop the community they have been living. Working as VHVs, they had a good experience working with others, whether the VHV’s network or external agencies they have to coordinate as a member of the Surveillance and Rapid Response Team (SRRT) under the supervision and coordination of the Department of Disease Control opens opportunities for VHVs’ self-development and learning from the training programs provided by the Ministry of Public Health (MoPH). Participating in the programs and community fieldwork could create a good relationship with the governmental agencies and VHVs’ network formally and informally.

### Local women VHVs and their engagement in community health during COVID-19

Since January 2020, VHVs has consistently played a role in preventing the spread of COVID-19. The main function is to screen individuals in each household, particularly those returning from other countries or high-risk areas in the country. They contact the subdistrict health-promoting hospital for the referrals of the patient if further treatment is needed. They also provide psychological support to people in the community to mitigate the psychological impact of the pandemic and provide medical supplies for critically ill patients, which are different from the services provided by medical personnel in hospitals. Door-to-door service reduces delivery and travel time, and this makes it easier operation in remote areas, coordinated by a communication platform used to communicate with each other, such as Line App between the VHVs, people in the community, and medical personnel. This increases the likelihood of referrals for treatment when symptoms are detected and surveillance for those in community quarantine. There is also support from local authorities, such as Sub-district Health Boards (SHBs) and central health agencies, to encourage VHVs to carry out their tasks. The pandemic has become the prioritized policy in every district; for this reason, various resources have been mobilized for the operation directly. The Subdistrict Health Fund has been used to strengthen the surveillance and health promotion capabilities of the VHV’s work. Telecommunication companies have also collaborated with the Ministry of Public Health to develop an online application used in local surveillance systems. The following are the dominant roles of local women as frontline health workers during COVID-19.

### VHVs as the role of community health caregivers

The role of VHVs before COVID-19 are community health caregivers in various fields and being trained for expertise in taking better care in the community in specific areas, such as the 12 fields of expertise for VHVs. From the training for expertise in particular fields, VHVs have attained the necessary and sufficient knowledge for providing primary healthcare to the community during the pandemic and could be applied very well in the context of COVID-19.

### VHVs as the surveillance and rapid response team (SRRT)

The Surveillance and Rapid Response Team (SRRT) is considered the main mechanism of the Department of Disease Control in dealing with the pandemic, which plays an important role in coping with the spread of the virus. Whether it is a pandemic in humans or in animals, VHVs have prior experience in dealing with pandemics, such as *SARS, Avian Influenza, Influenza, H1N1, etc.*; the VHVs as one of the team members in SRRT and are involved with the medical team as well as nurses in community hospitals and sub-district health promoting hospitals (SHPH), including Civil Defence Volunteers (CDVs) of the Ministry of Interior. The collaboration of many sectors in this is considered an essential factor for the prevention and surveillance of COVID-19. The VHV’s actions are to support the local community-level disease surveillance system based on an epidemiological protocol, such as proactive screening and timely reporting of infected people. They also assist medical personnel in taking care of community members during quarantine.

### VHVs as health facilitators and mediators

VHVs also play the role of health facilitators and mediators for patients with previously chronic NCDs such as diabetes, hypertension, and heart disease, which is a high-risk group that can cause severe symptoms after the infection of COVID-19. This is to prevent NCD patients, cases related to COVID-19, and other patients from not entering the hospital where they are the most at risk of infection. In addition, VHVs also play a role in coordinating between hospitals and patients in delivering the patient’s medicines, such as coordinating as a “grab drug” network that delivers medicines to homes for patients who need medication in times of the pandemic.

### VHVs as (acting) manager of community health funds and resources mobilization

VHVs, as managers of community health funds and resource mobilization, could assist local communities in managing district health funds and resource mobilization to prevent and control the spread of COVID-19. The VHVs play a role in the mobilization of resources during the pandemic in terms of donations, both in money and kinds of stuff and collective actions when necessary. In addition, VHVs is also an intermediary for coordinating funds and managing resources received from donation, and distributing resources to people who are in need. This is, for example, a person who is in-between quarantine, delivering food, or providing mental health assistance in neighbourhoods during difficult times.

## Discussion and conclusion

During COVID-19, Village Health Volunteers (VHVs) play an important role in health promotion, surveillance, prevention and control of the disease, restoration of health, and consumer protection. Previously, the performance of VHVs has been successful in primary health care and family planning that can increase the contraceptive rate and reduce the birth rate as targeted to reduce unwanted pregnancy, the incidence of HIV/AIDs infection, illness, mortality, and others. However, network partners and relevant agencies should improve the performance VHVs by focusing on the application of innovation and digital technology to health care to increase efficiency and effectiveness in local operations.

Based on data from fieldwork reveals that local women VHVs in Chiang Mai province are basically the villagers who have volunteered to work for their community. With the several advantages stance of the kinship relationship in rural society, they acknowledged leadership and trustfulness from the community. In general terms, frontline health workers include all types of health workers who provide care directly to their communities [5], but what makes VHVs, in this case, distinguished from other frontline health workers is that the motivation to participate in the program, do not about the financial matter but personal desire and other opportunities that create meaningful participation for a local woman based on her own interpretation. Generally, VHVs receive basic education and undergo necessary medical training or basic healthcare treatment. Beforehand, we assume that the VHVs’ characteristics are a “villager,“ which refers to ordinary community residents who are “low profile and unemployed.“ However, based on key informants’ Socioeconomic Status (SES), witnessed that some VHVs attained higher education, even graduate school, governmental officers, and business owners, and often held other positions in the village simultaneously with being VHVs. The main motivation is a personal desire to help others and do something for their community, including being accepted by the community they are in. Being women by nature, social expectations on the roles of women and personalities to be kind and gentle, caring for others, and able to take care of family members, and in these aspects, could be extended to take care of others in other families. Most VHVs have been growing up and living in the villages for a long time and are well-known to other community members. This resulted in good compliance with government measures passed on from VHVs to other community members. In this respect, the VHVs play a role as street bureaucrats [12–14] in helping the government, medical personnel, and community members; this is an essential factor in mitigating the COVID-19 pandemic at the local level.

Our study of women on the frontline of COVID-19 indicates that even though Thailand (herein referred to as Chiang Mai) is still a patriarchal society [15], however, playing the roles of VHVs of local women is perhaps one way to mobilize and empower women. As statically illustrated, women are the majority of the VHV population, which needs interpretation as to why it is what it is. What is the meaningful participation in community healthcare programs contributed to by local women VHVs? Based on intersectionality in our term mentioned earlier, community healthcare program participation of local women VHVs could empower and increase the role of women beyond the expectation of the countryside society only as a mother and wife; women are also expected as family caretakers to take care of the home and the well-being of family members. Performing as a VHV opens a window of opportunity for women to play a leading role in community health care and other roles. It can be seen from some of the VHVs has held positions as community (local political) leaders, such as village headmen, assistant village headmen (VHV-104, VHV-202, 204), and local politicians (Sub-district municipal council members) (VHV-201). Based on in-depth interviews, most of them used to serve as VHVs before, or even recently, they still serve as VHVs, along with other positions such as governmental officers, local leaders, and local politicians, as mentioned above.

In addition, our observations suggest that the motives or consequences of volunteering to take on the role of VHVs may be understood by a series of explanations on social-political networks. This can be seen that the VHVs take several roles and positions in their communities, and some of them have created a network with outside agencies as so-called *“wearing many hats”* simultaneously. Being VHVs allow them to get access to the position that they could create a social and local political network, both formal and informal. As a result, the VHVs are regarded as essential political action and opportunities; VHVs’ networks are closely tied with local politicians in the election campaign as election canvassers, or even the VHVs themselves, have moved into the political path. At the global level, gender challenges and equity in the health workforce are key to achieving universal health coverage by 2030 [7, 8]. Therefore, being local women, VHVs may be viewed as how to empower women’s roles to acquire knowledge, create capacity and self-confidence for women to participate in the development of their local communities, and further enhance the role of local women in other fields.

## Data Availability

This study used publicly available data, and the datasets used and/or analysed during the current study are available from the corresponding author upon reasonable request.

## List of abbreviations

VHVs: Village Health Volunteers
CDVs: Civil Defence Volunteers
CHWs: Community Health Workers
NCDs: Noncommunicable diseases
SRRT: Surveillance and Rapid Response Team
SHBs: Sub-district Health Boards
SHPH: Sub-district health-promoting hospitals
